# Intracerebral Administration of a Novel Self-Assembling Peptide Hydrogel Is Safe and Supports Cell Proliferation in Experimental Intracerebral Haemorrhage

**DOI:** 10.1007/s12975-023-01189-7

**Published:** 2023-10-18

**Authors:** Faye Bolan, Ben R. Dickie, James R. Cook, Josephine M. Thomas, Emmanuel Pinteaux, Stuart M. Allan, Alberto Saiani, Catherine B. Lawrence

**Affiliations:** 1 Geoffrey Jefferson Brain Research Centre, Manchester Academic Health Science Centre, Northern Care Alliance NHS Foundation Trust, The University of Manchester, Manchester, M13 9PT UK; 2 Division of Neuroscience, Faculty of Biology, Medicine and Health, The University of Manchester, Manchester, M13 9PT UK; 3 Division of Informatics, Imaging and Data Sciences, Faculty of Biology, Medicine and Health, University of Manchester, Manchester, M13 9PT UK; 4 Department of Materials, The University of Manchester, Manchester, M13 9PL UK; 5 Manchester Institute of Biotechnology, The University of Manchester, Manchester, M13 9PL UK

**Keywords:** Hydrogel, Intracerebral haemorrhage, Self-assembling peptide, Regenerative medicine Regeneration

## Abstract

Intracerebral haemorrhage (ICH) is the deadliest form of stroke, but current treatment options are limited, meaning ICH survivors are often left with life-changing disabilities. The significant unmet clinical need and socioeconomic burden of ICH mean novel regenerative medicine approaches are gaining interest. To facilitate the regeneration of the ICH lesion, injectable biomimetic hydrogels are proposed as both scaffolds for endogenous repair and delivery platforms for pro-regenerative therapies. In this paper, the objective was to explore whether injection of a novel self-assembling peptide hydrogel (SAPH) Alpha2 was feasible, safe and could stimulate brain tissue regeneration, in a collagenase-induced ICH model in rats. Alpha2 was administered intracerebrally at 7 days post ICH and functional outcome measures, histological markers of damage and repair and RNA-sequencing were investigated for up to 8 weeks. The hydrogel Alpha2 was safe, well-tolerated and was retained in the lesion for several weeks, where it allowed infiltration of host cells. The hydrogel had a largely neutral effect on functional outcomes and expression of angiogenic and neurogenic markers but led to increased numbers of proliferating cells. RNAseq and pathway analysis showed that ICH altered genes related to inflammatory and phagocytic pathways, and these changes were also observed after administration of hydrogel. Overall, the results show that the novel hydrogel was safe when injected intracerebrally and had no negative effects on functional outcomes but increased cell proliferation. To elicit a regenerative effect, future studies could use a functionalised hydrogel or combine it with an adjunct therapy.

## Introduction

Intracerebral haemorrhage (ICH) is a subtype of stroke that makes up around 10–15% of cases in Western countries. Although ICH is the deadliest form of stroke, current treatment options are limited and ICH survivors are often left with life-changing impairments resulting in a significant unmet clinical need and socioeconomic burden. All currently available interventions target aspects of primary injury, and there are no treatments that aim to protect damaged yet viable brain tissue or stimulate regeneration post ICH.

Novel regenerative medicine approaches are gaining interest in the treatment of stroke with the aim of repairing or regenerating lost tissue and recovering impaired function. Stroke is a viable target for regenerative medicine approaches for a number of reasons. Firstly, following acute brain injury, endogenous repair mechanisms, such as angiogenesis and neurogenesis, are activated and contribute to some restoration of function but are insufficient for the total repair of the damaged tissue [1–3]. Secondly, the resulting stroke lesion is thought to be relatively compartmentalised, which may allow a therapy to be delivered intracerebrally, direct to the site of injury, with minimal damage to adjacent viable tissue [1, 4]. The stroke lesion is also in direct contact with the perilesional tissue, which has the greatest potential for remodelling and repair [5–8].

Hydrogels are promising regenerative candidates for ICH, as they are designed to address multiple aspects of pathophysiology. For instance, hydrogel structure is designed to mimic the endogenous extracellular matrix (ECM), allowing them to act as a tissue scaffold and facilitate host cell infiltration into the lesion [9, 10]. Hydrogels can also act as delivery materials for other pro-regenerative therapies, which is proven to result in sustained, controlled release of the therapy over time at the site of injury, in contrast to bolus delivery [11, 12]. Only a few published studies have tested hydrogels in experimental ICH models, and the majority have focussed on acute administration time points after haemorrhage, using natural hydrogels (gelatin, keratin or a hyaluronic acid and chitosan blend), or the self-assembling peptide hydrogel (SAPH) RADA_16_ [13]. While the hydrogels tested in ICH models to date have some favourable regenerative properties [13], naturally derived hydrogels are associated with high batch-to-batch variability and potential immunogenicity [5], whereas some RADA_16_ SAPHs are acidic and lead to low levels of host cell infiltration when injected into the brain [14, 15]. Therefore, alternative hydrogel candidates are needed, to address the weaknesses of existing materials.

SAPHs are widely used in acute brain injury research due to their highly tunable properties (e.g. stiffness, porosity and degradation rate) and low immunogenicity [16, 17]. Another major advantage of SAPHs for central nervous system (CNS) regeneration is their injectability [18], which is important for minimally invasive administration to the brain. For application in brain repair, the ideal SAPH should mimic the mechanical properties of the brain, allow cell infiltration from the surrounding tissue, be retained at the injection site for long enough to facilitate regeneration, be non-immunogenic and eventually be degraded into safe by-products, in tandem with and at a similar rate to endogenous ECM deposition [19]. Novel SAPHs including Alpha2 have previously demonstrated good biocompatibility, low immunogenicity [18, 20] and improved survival and differentiation of cardiac [21] and neural progenitors in vitro [22]. However, the in vivo feasibility and safety of the Alpha2 SAPH as a regenerative biomaterial in an experimental disease model have not been explored, so the potential of Alpha2 for brain regeneration is unknown.

The aim of this study was to firstly determine the feasibility and safety of intracerebral injection of the novel SAPH hydrogel Alpha2 in a rat ICH model and secondly, to assess any therapeutic potential by studying its effect on functional and histological outcomes after ICH. Finally, to further explore the effects of the hydrogel, RNA sequencing was used to assess its effect on the transcriptional profile of the surrounding tissue after ICH. Overall, this study therefore represents the first application of this SAPH hydrogel for CNS regeneration.

## Methods

### Animals

Male Sprague–Dawley rats (340–550 g; Charles River, UK) were housed in groups of two to four under controlled housing conditions (temperature 21 ± 5 °C, humidity 55 ± 10%, 12-h light/dark cycle) with access to ad libitum food and water. All animal procedures were performed in line with the Animals (Scientific Procedures) Act (1986) and were approved by the local Animal Welfare and Ethical Review Body, University of Manchester, UK. All reporting of animal experiments complied with the ARRIVE guidelines (Animal Research: Reporting in In Vivo Experiments [23]). Prior to the start of any experimental procedures, all animals were handled for around 10 min per day for at least 5 days.

### Collagenase Model of ICH

ICH was induced using the collagenase model [24]. Briefly, anaesthesia was maintained with 2.5% isoflurane (in 30% O_2_:70% N_2_O). Collagenase (bacterial type-IV; 0.15U in 1 µl sterile saline, Sigma-Aldrich) was manually injected over one min using a glass micropipette into the right striatum (0.2 mm anterior, 3 mm lateral (right) and 5.5 mm from the dural surface, relative to bregma [25]). The micropipette was retracted after 10 min, and the wound was sutured. Cage mates were rehoused and allowed to recover from anaesthesia in a heated cabinet (27 °C). Softened food pellets were given for the first 3 days post-surgery. No deaths occurred after ICH.

### Intracerebral Administration of Hydrogel

Alpha2 hydrogel (PeptiGel®; elastic modulus 1.7±0.2 kPa) and a modified FITC-conjugated Alpha2 hydrogel (elastic modulus 1.8±0.2 kPa) were used (Cell Guidance Systems, UK). Seven days after induction of ICH, rats were re-anaesthetised, and after positioning in a stereotaxic frame, a gastight 50-µl Hamilton syringe (1800 series) was positioned on the dura, at the centre of the burr-hole, and the intervention (hydrogel or vehicle) was stereotaxically injected at a pre-determined depth (see below) at a rate of 10 µl/min. The needle was held in place for a further 10 min to prevent reflow, and animals were recovered from surgery as previously described. Hydrogels were sterilised in a UV chamber for 10 min prior to administration.

An initial study was performed to track the location and retention of the hydrogel at the injection site over a 14-day period by using a modified FITC-conjugated Alpha2 hydrogel. Rats (*n* = 4) received stereotaxic injection of FITC-hydrogel (25 µl) 7 days after ICH. Rats were then culled at 1 (8 days post-ICH), 3 (10 days post-ICH), 7 (14 days post-ICH) and 14 days (21 days post-ICH) after hydrogel injection (*n* = 1/time point).

**Fig. 1:**
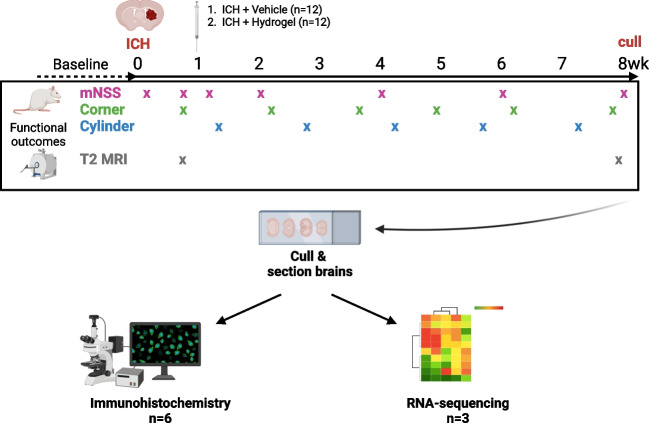
The effect of hydrogel administration on ICH outcomes. Intracerebral haemorrhage (ICH) was induced in male Sprague–Dawley rats and 7 days later, either vehicle (*n* = 12), or Alpha2 hydrogel (*n* = 12) was administered stereotaxically into the haemorrhage site. The modified neurological score (mNSS), corner and cylinder functional tests were carried out over an 8-week period. To study anatomical disease features and lesion volume a T2 MRI sequence was performed at 6 days (pre-intervention) and 8 weeks after ICH. After 8 weeks, animals were culled, and brains taken for RNA-sequencing (*n* = 3/group) or immunohistochemistry (*n* = 6/group)

In a separate study (Fig. 1), vehicle (20 µl vehicle, *n* = 12) or hydrogel (20 µl of Alpha2 hydrogel, *n* = 12) was injected on day 7 after ICH. Hydrogel injection volume was two-thirds of the average haematoma volume at day 6 post-ICH (determined by T2 magnetic resonance imaging [MRI]) of the first batch of animals in the study (*n* = 6). Rats were excluded from the study if the day 6 ICH MRI volume was < 20 mm^3^. The depth co-ordinate for injection was also determined for each animal by the T2-weighted MR scan on day 6 post-ICH, where the centre of the haematoma was defined as the injection depth (between 5.5 ± 0.5 mm from the dural surface). Rats were culled 8 weeks (56 days) after ICH induction.

As the greatest variability will be produced by behavioural data, a power analysis was performed (G-Power) using previously acquired data from the corner test (average turns towards the affected side, 0.12 ± 0.12 [SD]) and significance level (*α*) of < 0.05, power (*β*) of 0.8 and an effect size of 0.2 (i.e. a 20% change in turns to the affected side). As corner test data were non-parametric, a 15% increase was applied, and an additional three animals per group were added to account for attrition due to dropout/exclusion criteria in the functional tests, resulting in a final *n* number of 12 rats per group. All animals were allocated/stratified to an experimental group on day 6 post-ICH based on the haematoma volume on that day (determined by MRI), to ensure average haemorrhage sizes were comparable between treatment groups. After each MRI scanning day, all animals from that block were ranked based on their haematoma volume, and an independent experimenter then allocated the animals to one of the treatment groups (hydrogel or vehicle). Treatment allocation concealment was not possible due to the clear differences in the hydrogel and vehicle administration (e.g. due to viscosity, transparency of the treatments and a single experimenter conducting surgeries). Cages were blinded after surgery by an independent experimenter, and all data analyses were performed in a blinded manner.

### Magnetic Resonance Imaging

MRI imaging was performed using a T2-weighted sequence to determine haematoma, hemisphere and ventricle volume using a 7-Tesla, horizontal-bore magnet (Agilent Technologies, Oxford, UK) connected to a Bruker Biospec Avance III console (Bruker Biospin, Coventry, UK). Anaesthesia was induced and maintained using 5% or 2% isoflurane respectively (in 30% O_2_:70% N_2_O). A three-plane localiser scan was performed to ensure the correct positioning of the animal in the magnet, followed by a high spatial resolution T2-TurboRARE weighted sequence using the following parameters: repetition time/echo time = 3192/33 ms; number of excitations = 5; field of view = 35 × 35 mm^2^; matrix size = 512 × 512 mm; slice thickness = 0.5 mm; number of slices = 30. After scanning, animals were allowed to recover from anaesthesia in a heated chamber (27°C) and then returned to home cages. To measure haematoma, hemisphere and ventricle volumes, regions of interest (ROI) were manually drawn on T2-weighted image slices in ImageJ (v1.49, NIH, Bethesda, MD), and volumes were determined by calculating the area under the curve. To calculate the percentage of tissue atrophy for the ipsilateral (ICH-affected) hemisphere, ventricle volumes were first subtracted from the total ipsilateral and contralateral hemisphere volumes and the percentage of tissue atrophy was then calculated relative to the contralateral hemisphere.

### Functional Outcome Measures

Several tests of functional outcome were used to assess severity and recovery after ICH. Animals were acclimated to testing rooms for at least 1 h prior to testing, which was performed in the same environment throughout.

#### Modified Neurological Severity Score

The 18-point modified neurological severity score (mNSS) or ‘neuroscore’ was performed at baseline and days 1, 5, 8, 14, 28, 42 and 56 after ICH. A point was given when the animal was unable to perform a task or a reflex was absent (scores of 1–6 represented mild injury, 7–12 moderate injury and 13–18 severe injury).

#### Corner Test

Rats were placed facing forward in a corner (30° angle) made from two adjoined sheets of clear Perspex (H30 cm × W42 cm). The turning direction was recorded for 10 consecutive trials, and a percentage of turns towards the stroke-affected side was calculated. Baseline measurements were the average of two pre-ICH trials on consecutive days, and the trial was repeated on days 5, 15, 25, 35, 45 and 55 post-ICH. Animals with a baseline turning preference for one side of ≤ 30% or ≥ 70% were excluded for the remainder of the experiment for this test, as this demonstrated a pre-existing limb-use bias [26, 27].

#### Cylinder Test

Rats were placed into a Perspex cylinder (H30 cm × 19.4 cm internal diameter) positioned on top of a clear Perspex sheet with a Panasonic camcorder below and left to explore for 6 min. For scoring, rearing movements initiated with either the left, right or both paws were recorded, and the percentage use of the impaired limb was calculated. Baseline measurements were the average of two pre-ICH trials on consecutive days, and the trial was repeated on days 10, 20, 30, 40 and 50 after ICH. Animals performing less than 10 rears were excluded for that time point only, and animals with a baseline paw-use preference for one side of ≤ 30% or ≥ 70% were excluded for the remainder of the experiment for this test, as this demonstrated a pre-existing limb-use bias [26, 27].

### Tissue Processing for FITC Hydrogel Imaging

Rats were culled by an overdose of CO_2_ followed by cervical dislocation, and brains were snap-frozen and cryosectioned at 20 µm. To determine the location and retention of the hydrogels and explore whether host cells from the surrounding tissue could migrate into the material, sections were stained with haematoxylin and eosin (H&E) [28].

### Immunohistochemistry

Under anaesthesia (5% isoflurane), vehicle- and hydrogel-treated animals (*n* = 6/group) were perfused transcardially with 0.9% ice-cold saline, followed by 4% paraformaldehyde (PFA). Brains were removed and post-fixed in 4% PFA solution for 24 h and transferred to a 20% cryoprotectant sucrose solution for a further 24 h. The brains were then embedded in an optimal cutting temperature compound, flash-frozen in − 50 °C isopentane and stored at − 80 °C until further processing. Brains were sectioned coronally (at 20 µm) using a Leica CM3050 cryostat (Leica Biosystems, UK) and stored at − 20 °C until required. Immunohistochemistry was performed using the following primary antibodies: goat anti-GFAP (for astrocytes; 1:1000, Abcam), rabbit anti-Iba1 (for microglia/macrophages; 1:2000, WAKO), rabbit anti-collagen IV (for blood vessels; 1:300, Abcam), rabbit anti-doublecortin (DCX, neuroblasts; 1:500, Abcam), rabbit anti-Ki67 (for proliferating cells; 1:500, Abcam) and rabbit anti-β3-tubulin (for immature neurons; 1:500, Abcam).

For all immunohistochemistry protocols, antigen retrieval was performed by placing slides in a 97.5 °C water bath in either Tris–EDTA (pH 8.6) or Na-Citrate (pH 6.0) solution. Non-specific binding was blocked by incubating slides with either 2% bovine serum albumin (BSA) or 2% normal serum (NXS) from the species in which the secondary antibody was raised, for 1 h. Primary antibodies were diluted (in PBS/1% BSA/NXS, 0.3% Triton X-100 and 0.1% tween) and incubated on slides overnight at 4 °C. Slides were then washed (PBS with 0.1% tween). For GFAP/Iba1 and β3-tubulin, slides were then incubated for 1 h with fluorescently-conjugated secondary antibody diluted in PBS with 0.1% BSA/NXS and 0.1% tween (GFAP, anti-goat 647; Iba1, anti-rabbit 555; β3-tubulin, anti-rabbit 647; all Alexa Fluor®, Invitrogen). Slides were washed and nuclei counterstained by incubation in 4′,6-diamidino-2-phenylindole (DAPI, 1 μg/mL). Slides were cover-slipped with Prolong™ Gold anti-fade reagent.

To reduce the effect of autofluorescence from haemosiderin in macrophages, signal amplification was performed for some antibodies using a Tyramide Kit (Alexa Fluor™ 555 Tyramide SuperBoost™ Kit, streptavidin, ThermoScientific) following the manufacturer’s instructions. For collagen IV after primary antibody incubation, endogenous peroxidase activity in the tissue was blocked with 0.3% H_2_O_2_ for 30 min. Slides were then washed in Tris-buffered saline with 0.1% tween (TBST). An anti-rabbit biotinylated secondary antibody (Vector Labs) was then incubated for 1.5 h, diluted in TBST with 0.1% BSA/NXS. After further washes, the slides were incubated with a streptavidin-horse radish peroxidase tertiary antibody for 45 min. Tyramide reaction buffer was then incubated on slides for 8 min. To further reduce the effect of autofluorescent blood pigments, the TrueView autofluorescence quenching kit was used.

The tyramide protocol was also used for double labelling for DCX/Ki67, as both antibodies were raised in the same species. Here, Ki67 was tyramide amplified (as described above), then a second antigen retrieval and the standard immunohistochemical protocol were performed (as above) for DCX, and an anti-rabbit 647 secondary antibody (Alexa Fluor®, Invitrogen) was used.

Negative control slides were prepared to help distinguish specific labelling from autofluorescence where all immunohistochemistry steps were performed in the same way, except that no primary antibody was added in the primary antibody buffer.

### Immunohistochemistry Image Analysis

Images were collected using either an upright fluorescence microscope (Zeiss Axioimager.D2) or slide-scanner (3D-Histech Pannoramic-250 microscope slide-scanner). All image analysis was carried out blind to the experimental group, and quantification parameters were constant for the whole set of images for each marker.

#### DCX/Ki67 Quantification

A DCX (neuroblasts) and Ki67 (proliferating cells) co-label was performed to measure neural progenitor cell recruitment from the lateral ventricles and the number of actively proliferating cells in the lesion. For DCX, a region of interest (ROI) covering the lateral ventricles and superior edge of the lesion (3 per brain) and a size- and region-matched control ROI from the contralateral hemisphere (3 per brain) was drawn and exported to ImageJ, and mean integrated density was calculated. Data are presented as the fold-change in expression from the contralateral hemisphere. For Ki67, ROIs comprising the entire lesion (3 per brain) were drawn. The positive cell detection tool in QuPath was used to calculate the number of Ki67 positive cells, as a percentage of total DAPI-stained nuclei in the region.

#### GFAP/Iba1 Quantification

A GFAP and Iba1 co-label was used to quantify glial scar formation and to estimate microglial/macrophage activation and recruitment around the site of injury. To quantify the expression of both markers, 3 ROIs were drawn in areas corresponding to the superior, lateral and inferior aspects of the lesion border (in 3 sections per animal). A size- and region-matched area in the contralateral striatum was taken as control tissue. No quantification of GFAP/Iba1 was performed within the haemorrhage lesion itself due to the intense auto-fluorescence signal (and therefore channel bleed through) from haemosiderin-containing macrophages/microglia, which is a common feature of ICH tissue. The mean integrated density was calculated in ImageJ. Data are presented as the fold-change in marker expression from the contralateral hemisphere.

#### Collagen IV Quantification

Collagen IV was chosen to quantify blood vessel density in the lesion border as a measure of angiogenesis/vascularisation. As the collagen IV stain was used to gauge angiogenesis, it was not quantified in the lesion interior, due to complex labelling in this region that did not always appear to be blood-vessel associated. ROI of interest were drawn (as described for GFAP/Iba1) in ImageJ, the background signal was subtracted and brightness/contrast settings were auto-adjusted. ROIs were then imported into REAVER software (Rapid Editable Analysis of Vessel Elements Routine, https://github.com/uva-peirce-cottler-lab/public_REAVER), and a threshold grey value of 0.045 was applied. Vessel parameters (area, length density and number of branchpoints) were automatically quantified in REAVER, and data are presented as the fold-change compared to the contralateral hemisphere.

### RNA-Sequencing (RNA-seq)

Haemorrhage tissue was taken from the ipsilateral striatum of rats 8 weeks after vehicle or hydrogel, and the contralateral striatum of vehicle-treated rats was taken as control (*n* = 3/group). Total RNA was isolated (QIAGEN RNeasy® lipid tissue mini kit), and its quality and integrity were assessed using a 4200 TapeStation (Agilent Technologies) before libraries were generated using the Illumina® Stranded mRNA kit (Illumina, Inc.) according to the manufacturer’s protocol. Briefly, total RNA (typically 0.025–1 µg) was used as input material from which polyadenylated mRNA was purified, and then mRNA was fragmented under elevated temperature and reverse-transcribed into first strand cDNA using random hexamer primers. Second-strand cDNA was then synthesised to yield blunt-ended, double-stranded cDNA fragments (to prepare for dual indexing). A PCR amplification step was then used to add index adapter sequences to create the final cDNA library. The adapter indices enabled the multiplexing of the libraries, which were pooled prior to cluster generation using a cBot instrument. The loaded flow cell was then paired-end sequenced (76 + 76 cycles, plus indices) on an Illumina HiSeq4000 instrument. Finally, the output data was demultiplexed, and BCL-to-Fastq conversion was performed using Illumina’s bcl2fastq software, v2.20.0.422.

For analysis, unmapped paired-end sequences were tested by FastQC v0.11.3 (http://www.bioinformatics.babraham.ac.uk/projects/fastqc/) and FastQ Screen v0.13.0 (https://www.bioinformatics.babraham.ac.uk/projects/fastq_screen/). Gene reads were trimmed to remove any adapter or poor-quality sequence using Trimmomatic v0.39 [29]. Filtered gene reads were mapped to the rat reference genome sequence (rn6) [30], using STAR v2.7.7a [31]. The genome index was created using the Ensembl v104 ‘Rattus_norvegicus.Rnor_6.0.104’ gene annotations, with a flag suitable for read length applied (–sjdbOverhang 75) [32]. During mapping, the flags ‘–quantMode GeneCounts’ were used to generate read counts per gene. Normalisation of read counts and differential expression analysis was performed using DESeq2 v1.30.1 [33] on R v4.0.4 (R Core Team 2021). Log fold change shrinkage was applied using the lfcShrink function along with the ‘apeglm’ algorithm [34]. Differentially expressed genes (DEG) between groups were identified using a Log2 fold-change > 0.5 and a false discovery rate (FDR)-adjusted *p* value (padj) of < 0.05. K-means clustering was conducted on the DEGs using the elbow method [35]. The EnrichR R package was used for gene-set enrichment, by querying the EnrichR platform [36]. Ingenuity pathway analysis (IPA, QIAGEN) was also was performed on the filtered DEGs to infer the activation of canonical pathways. Only the top five canonical pathways with network bias-adjusted *p* values of < 0.05 were included.

### Statistical Analysis

Unless otherwise stated, data were analysed in GraphPad Prism v9.0 (GraphPad). Data are presented as mean ± SD or median ± interquartile range (IQR) as stated. The D’Agostino-Pearson (*n* ≥ 8) or the Shapiro–Wilk (*n* = 3–7) normality tests were used to identify parametric and non-parametric datasets. To compare two datasets (single time point), an unpaired Student’s *t*-test (parametric) or Mann–Whitney test (non-parametric) was chosen. For data with two factors (time and treatment), a two-way ANOVA (with Sidak’s multiple comparisons test) was used. For repeated measure analysis, a mixed-effects analysis with Geisser-Greenhouse (followed by Sidak’s multiple comparisons test) was performed. *p* < 0.05 was considered significant.

## Results

### FITC-Conjugated Alpha2 Hydrogel Remains In Situ for At Least 14 Days and Allows Host Cell Infiltration

**Fig. 2:**
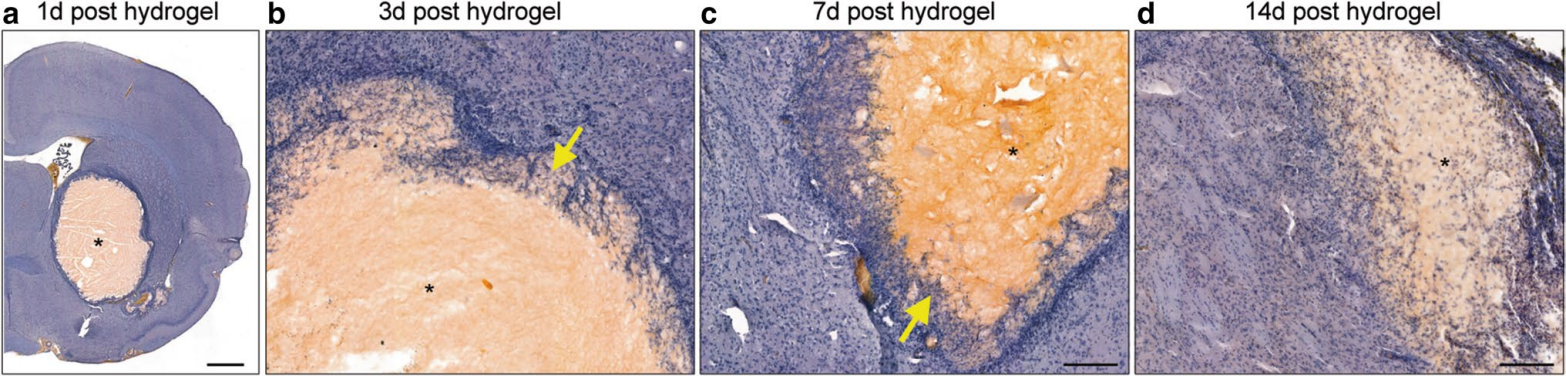
Alpha2 hydrogel supported cell infiltration. FITC-conjugated hydrogel was stereotaxically injected 7 days after intracerebral haemorrhage (ICH) and rats culled at 1, 3, 7 and 14 days later, and coronal sections stained with haematoxylin and eosin. The hydrogel appeared bright orange by brightfield microscopy (*) and was retained at the injection site for at least 14 days. **a** There was minimal cell infiltration at 1 day (8 days post-ICH). **b** At 3 days (10 days post-ICH) and **c** 7 days (14 days post-ICH) after injection, there was some degree of host cell infiltration (purple cells, yellow arrows) at the border of the hydrogel. **d** By 14 days (21 days post-ICH), cells had infiltrated to the centre of the hydrogel indicating that host cells could migrate through the porous hydrogel structure. Scale bars, **a** 1 mm, **b**–**d** 200 µm

The FITC-conjugated Alpha2 hydrogel was used to track its retention and location over a period of 14 days after administration (7 days post-ICH). H&E staining showed that the hydrogel remained in situ for at least 14 days and endogenous cells from the adjacent tissue infiltrated into the hydrogel after injection (Fig. 2). One day after injection, a few cells were observed in the hydrogel (Fig. 2a). At 3 days post-injection, cells from all regions of tissue in contact with the material had migrated into the implant, and the surrounding healthy tissue appeared normal (Fig. 2b). By 7 days, host cells moved further into the hydrogel, and by 14 days, cells were found at the centre of the material (Fig. 2c–d).

### Alpha2 Hydrogel Had No Effect on the Lesion Volume or Tissue Atrophy at 8 Weeks

**Fig. 3:**
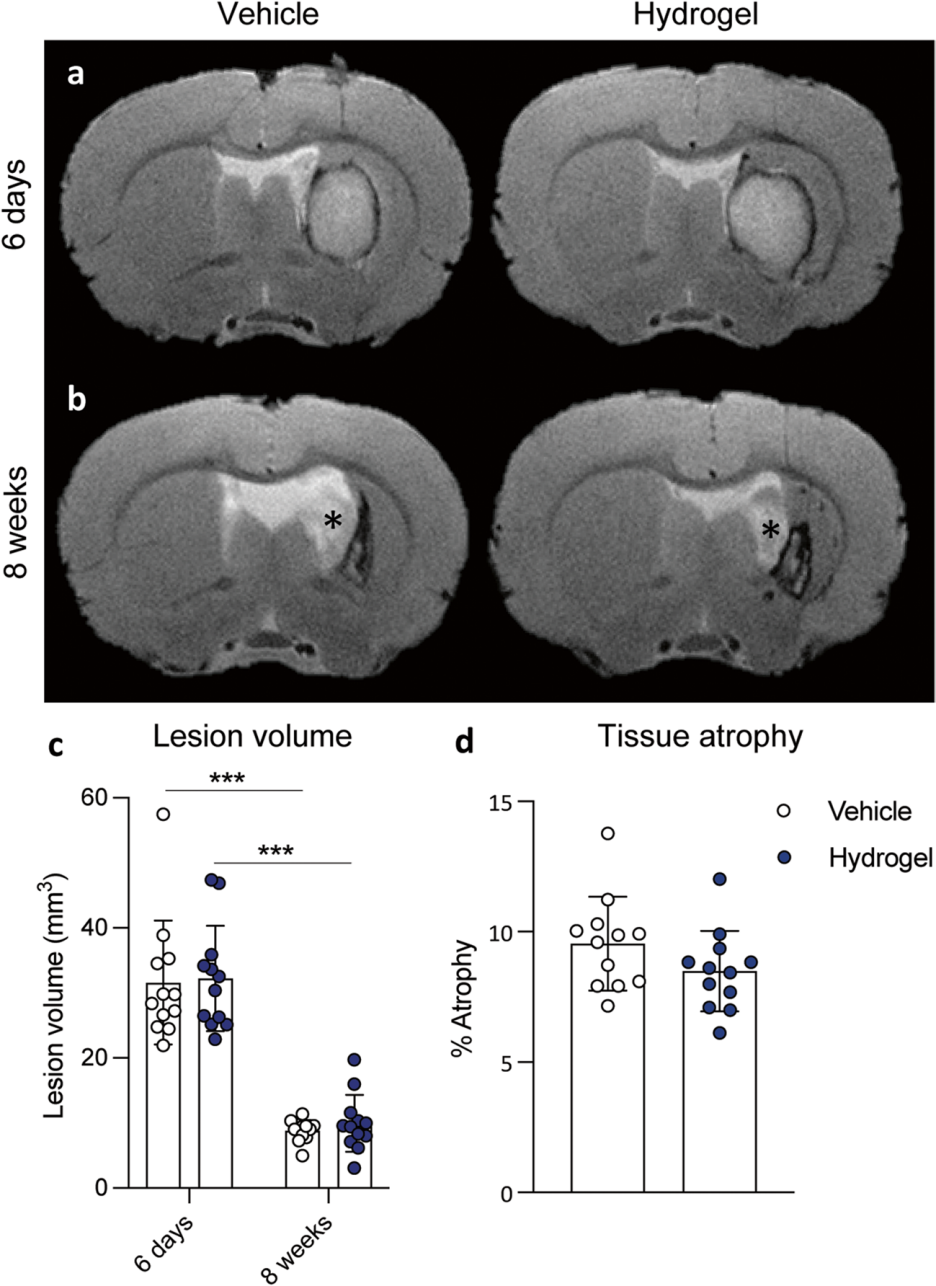
Hydrogel had no effect on lesion volume or tissue atrophy at 8 weeks. **a** A T2-weighted magnetic resonance imaging showed moderate striatal haematomas were present 6 days after induction of intracerebral haemorrhage (ICH) (pre-intervention). **b** Eight weeks after vehicle or hydrogel administration, there were no discernable differences in lesion appearance and no remaining hydrogel was visible but, marked ventricle enlargement (*) was present in both groups. **c** Prior to intervention, the 6-day lesion volumes were comparable for the vehicle and hydrogel groups and, no effect of hydrogel was seen at 8 weeks after injection. **d** No significant difference in the degree of tissue atrophy after 8 weeks. Data are mean ± S.D., *n* = 12/group. **c** Two-way ANOVA with Sidak’s test for multiple comparisons, or **d** unpaired Student’s *t*-test. ***p < 0.001 versus 6 days

At 6 days post-ICH, the haematoma was clearly visible on T2-weighted MRI images. In both groups, a rim of dark or hypointense T2 signal surrounded a core of hyper-intense signal observed in both groups (Fig. 3a). At 8 weeks after ICH-marked ventricular enlargement was present in both vehicle- and hydrogel-treated groups (Fig. 3b), and an area of hypo-intense signal containing hyper-intense regions indicating the lesioned tissue was observed. No qualitative differences in these parameters were observed between vehicle and hydrogel groups, and no remaining hydrogel was visible.

Prior to the intervention, at 6 days post-ICH, the average lesion volume measured by T2-weighted MRI was similar for the vehicle and hydrogel groups (Fig. 3c). By 8 weeks post-ICH, there was a significantly smaller lesion compared to 6 days (*p* < 0.001 for both groups, Fig. 3c), but there was no significant difference between treatment groups at either time point. The amount of tissue atrophy 8 weeks after ICH was not different between the groups (Fig. 3d).

### Alpha2 Hydrogel Had No Detrimental Effects on Behavioural Outcomes

**Fig. 4:**
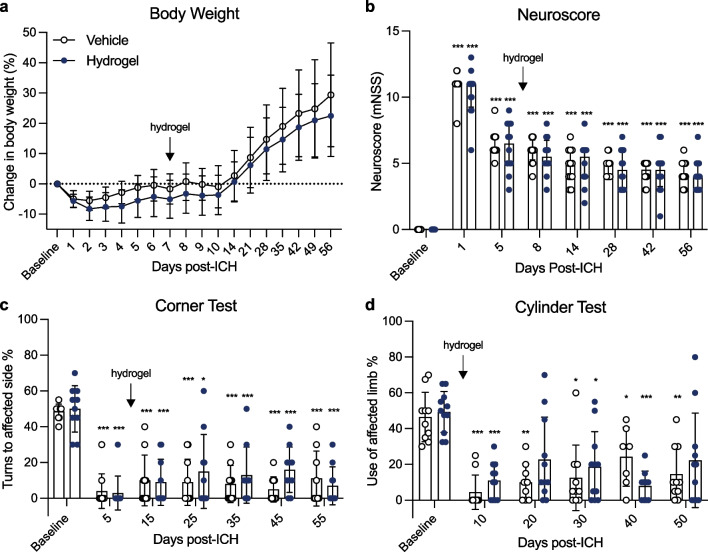
Hydrogel administration has no detrimental effect on functional outcomes. **a** All animals lost weight following intracerebral haemorrhage (ICH), before recovering to baseline weight. There was no significant difference in body weight between groups, at any time point. **b** There was a significant increase in median neuroscore at all time points after ICH compared to baseline but no difference between treatment groups. **c** In the corner test, turning towards the stroke-affected side significantly reduced after ICH but there was no difference between experimental groups, at any time point. **d** Use of the affected limb in the cylinder test significantly decreased after ICH in both groups, with no difference observed between treatment groups. Experimental groups/time points compared by mixed-effects model with Sidak’s test for multiple comparisons. Data are **a**, **c**, **d** mean ± S.D. and **b** median ± IQR. *p < 0.05, **p < 0.01, ***p < 0.001 versus baseline for the respective treatment group

To test for welfare and assess if the hydrogel had any impact on animal behaviour, body weight and several functional outcome tests were analysed up to 8 weeks after injection. There was a significant effect of time on change in body weight after ICH compared to baseline (*p* < 0.001; Fig. 4a). For the vehicle group, body weight change significantly increased from baseline at all time points after day 21 (*p* < 0.001). Body weight in the hydrogel group was significantly less from baseline values at days 2–4 (*p* < 0.01) post-ICH and increased at all time points after 28 days (*p* < 0.001). There was no statistically significant difference between the treatment groups at any time point.

In the neuroscore test, there was a significant overall effect of time (*p* < 0.001), and performance in both treatment groups at all time points was significantly different from baseline values (all *p* < 0.001; Fig. 4b). Performance in the neuroscore test was not significantly different at any time point between treatment groups.

In the corner test, there was a significant reduction in percentage turns to the affected side (overall effect of time *p* < 0.0001) after ICH, compared to baseline at all time points, and there was minimal recovery of turning behaviour over the course of the 8-week study, in both groups (Fig. 4c). There were no statistically significant differences in corner performance between the treatment groups, at any time point.

In the cylinder test, the use of the affected (left) limb for initiating rearing movements was significantly reduced at most time points after ICH compared to baseline in both treatment groups (*p* < 0.05–0.001; Fig. 4d), but with no difference between treatment groups.

### Proliferating Neuroblasts Were Found Within the Lesion of Both Groups, with Significantly More Proliferating Cells After Alpha2 Hydrogel Injection

**Fig. 5:**
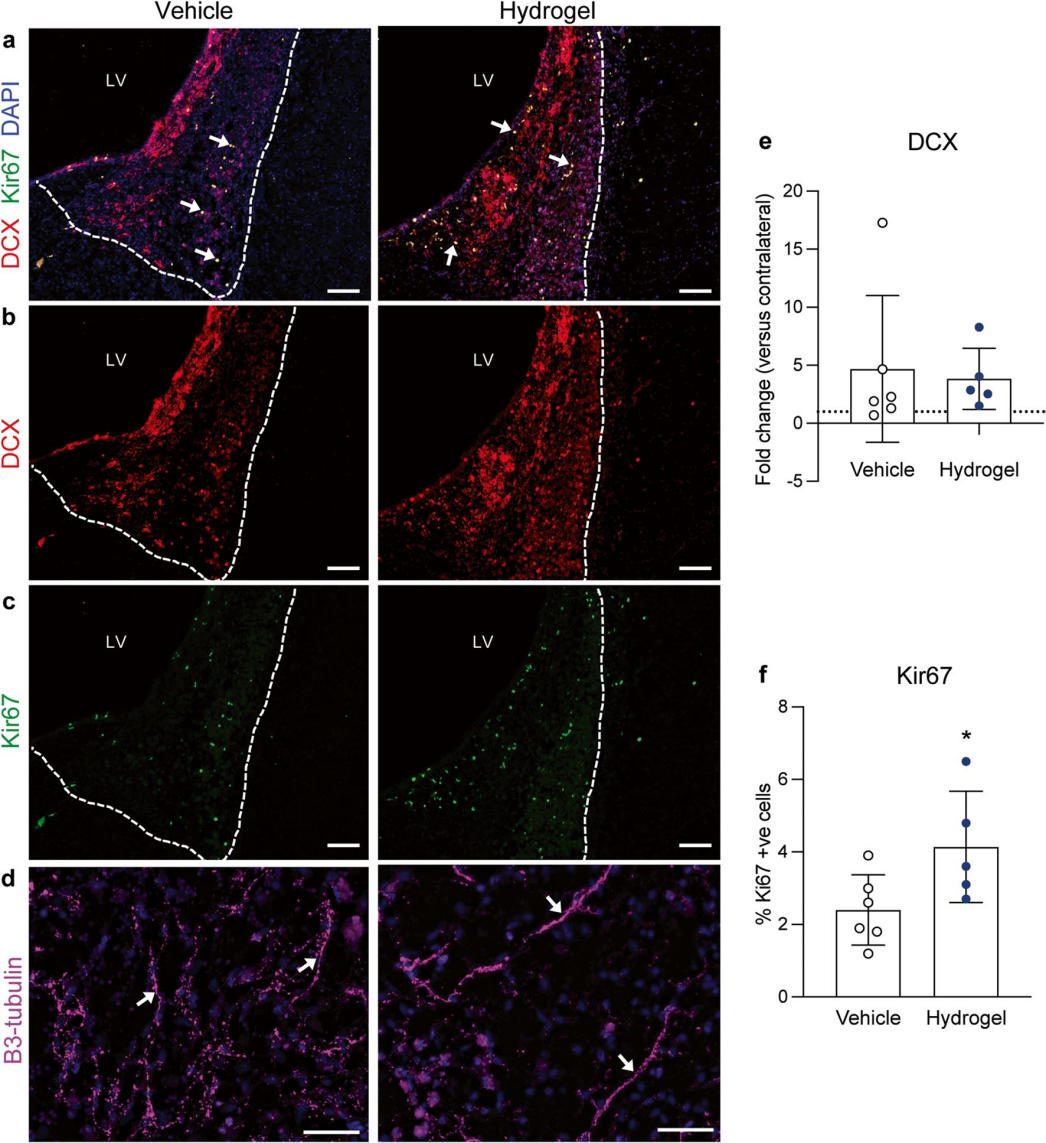
Proliferating cells increase after Alpha2 hydrogel injection. **a** Co-labelling of doublecortin (DCX; red) and Ki67 (green) revealed many instances of co-expression (white arrows), indicating the presence of proliferating neuroblasts in the lesion (white dashed line), in both treatment groups, 8 weeks after intracerebral haemorrhage (ICH). **b** Intense DCX expression was seen at the lateral ventricle (LV) walls and throughout the lesion in both groups. **c** There appeared to be more Ki67 + cells in and around the lesion of the hydrogel group, compared to vehicle. **d** Expression of the immature neuronal marker β3-tubulin (pink) was observed throughout the ICH lesion, where long microtubule networks had formed (white arrows). **e** There was no difference between the groups in the fold change expression of DCX, compared to the contralateral hemisphere. **f** There was a significant increase in the proportion of Ki67 + cells in lesion in the hydrogel group compared to the vehicle group. N.B. there was a processing error for Ki67 for *n* = 1 in the hydrogel group. Data are mean ± SD. *n* = 5–6 **e** Mann–Whitney test, **f** unpaired t-test. *p < 0.05. LV, lateral ventricle. Scale bar (**a**–**c**) = 100 µm, (D) = 50 µm

Labelling of DCX and Ki67 demonstrated many instances of co-labelling within the lesion of both groups, indicative of actively proliferating neural progenitors at the site (Fig. 5a). DCX labelling was particularly intense at the wall of the lateral ventricles that bordered the lesion (Fig. 5b), whereas Ki67 labelling was distributed throughout the lesion (Fig. 5c). Labelling with a βIII-tubulin antibody revealed networks of immature neurones at the core of the lesion in both groups after 8 weeks, indicative of a neurogenic response (Fig. 5d). Quantification of the fold-change in DCX expression (from the contralateral hemisphere) found no difference between the experimental groups at 8 weeks (Fig. 5e). For Ki67, there was a significant increase (73%) in the percentage of Ki67-positive cells in the lesion at 8 weeks in the hydrogel group, compared to vehicle-treated animals (*p* < 0.05, Fig. 5f).

### Alpha2 Hydrogel Had No Effect on the Astrocyte or Microglial Response After ICH

**Fig. 6:**
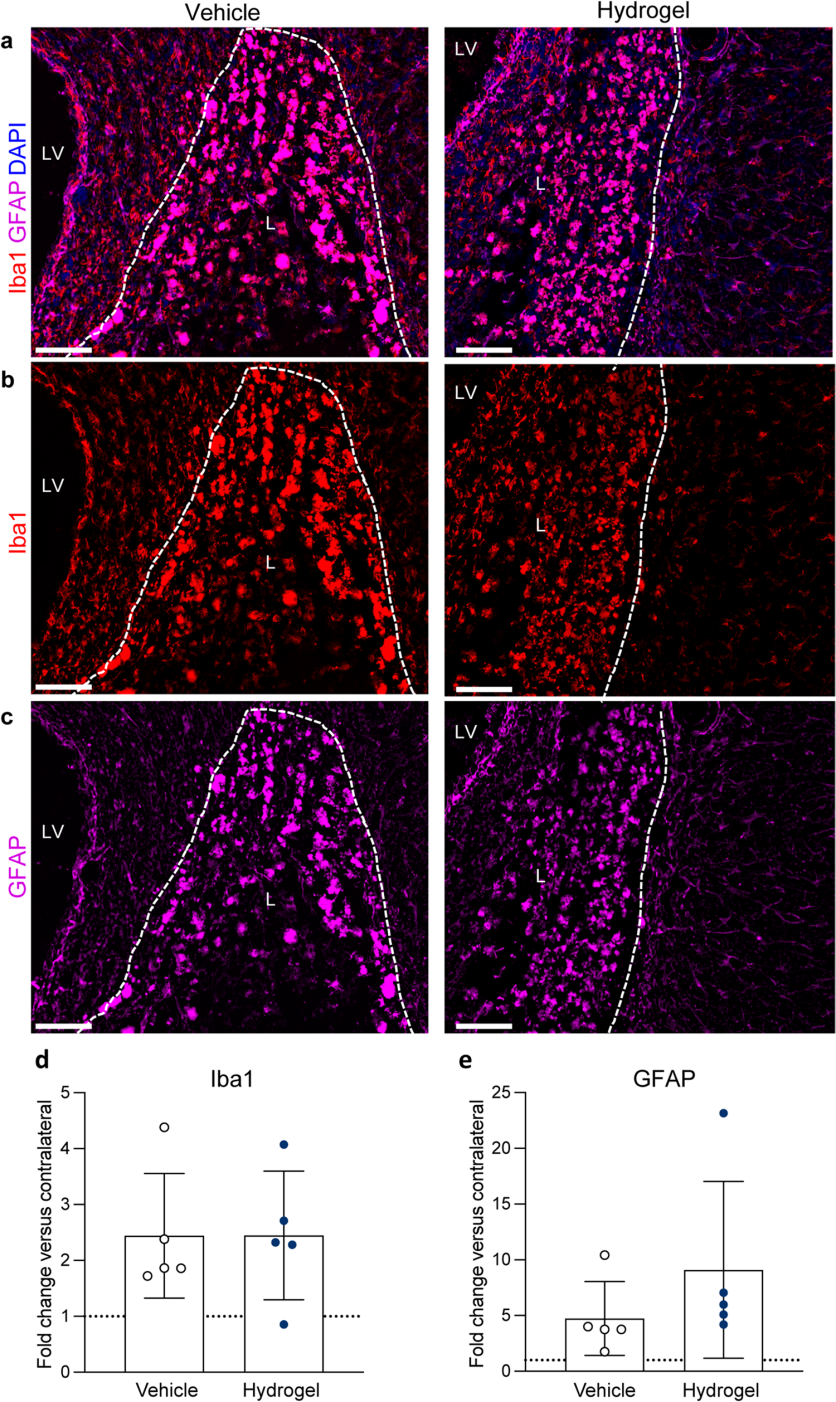
Hydrogel administration had no effect on microglia or astrocyte response. **a** Iba1 (red) and GFAP (pink) co-labelling did not reveal signs of a prominent glial scar at 8 weeks post-intracerebral haemorrhage (ICH). Strongly autofluorescent haemosiderin-containing macrophages/microglia can be seen in the lesion (L), which were also detected when visualising GFAP, due to signal bleed-through. Outside of the lesion border (white dashed line), there were no clear differences in morphology or intensity of **b** Iba1 (macrophage/microglia) or **c** GFAP (astrocyte) staining, between treatment groups. Quantification at the lesion border of **d** Iba1 found increased expression in the stroke-affected hemisphere compared to contralateral (> onefold change expression) in the vehicle (*p* < 0.05) and hydrogel (*p* < 0.01), and for **e** GFAP in the hydrogel groups (*p* < 0.01), but no difference between the treatment groups. Scale bar = 100 µm. LV, lateral ventricle. Data are mean ± S.D., *n* = 5/group. Mann–Whitney test. N.B. tissue was damaged during processing for *n* = 1 animal in each group

Iba1 and GFAP co-labelling 8 weeks after ICH found that hydrogel administration did not appear to have an effect on glial scarring or microglial/macrophage response at the lesion border, where quantification was performed (Fig. 6a–c). Macrophages/microglia within the lesion itself were intensely labelled due to specific Iba1 labelling and non-specific autofluorescence from haemosiderin within these cells; this non-specific autofluorescent signal was also observed in the lesion core when visualising GFAP. Quantification of both markers at the lesion border revealed significant increases in Iba1 expression versus the contralateral hemisphere in both groups (vehicle, *p* < 0.05; hydrogel, *p* < 0.01), and a significant increase in GFAP versus the contralateral hemisphere in the hydrogel group (*p* < 0.01). There was no significant difference between treatment groups in the fold change (from the contralateral hemisphere) in Iba1 (Fig. 6d) or GFAP expression (Fig. 6e).

### Alpha2 Hydrogel Had No Effect on Collagen IV Expression After ICH

**Fig. 7:**
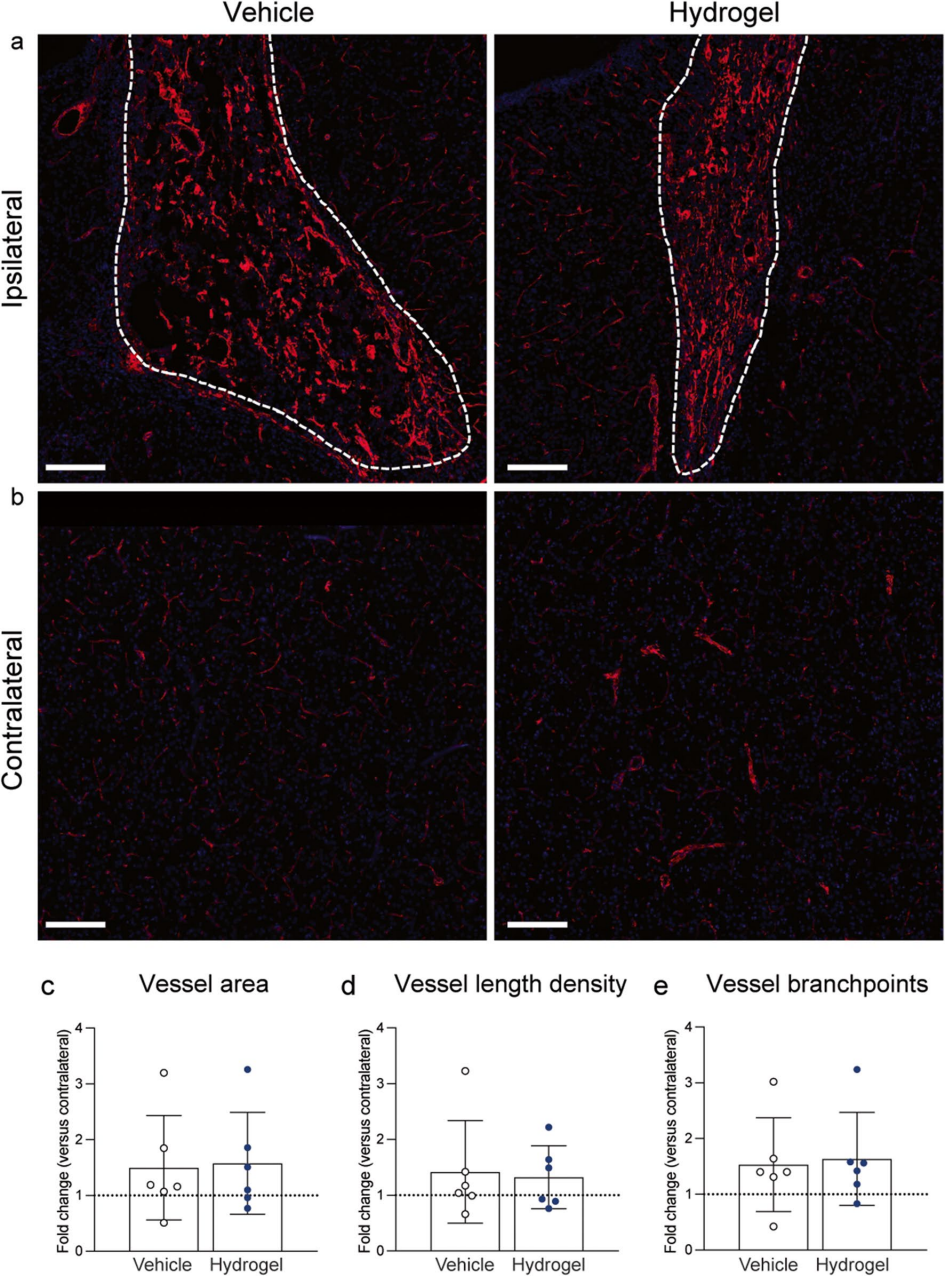
Upregulation of the vessel marker collagen IV in the lesion after intracerebral haemorrhage (ICH) but no effect of hydrogel administration. **a** Intense collagen IV labelling is present in the lesion (white dashed line) of both vehicle and hydrogel groups, when compared to **b** the contralateral hemisphere. **c**–**e** Quantification of vessel parameters at the lesion border found no difference in the fold change expression of collagen IV (from contralateral hemisphere) in **c** vessel area, **d** vessel length density or **e** the number of branchpoints, between vehicle and hydrogel groups. Scale bar = 200 µm. Mean ± S.D., *n* = 6/group. Mann Whitney test

A clear increase in the intensity of collagen IV labelling in the lesion core 8 weeks after ICH was observed (Fig. 7a) when compared to the surrounding tissue and the contralateral hemisphere (Fig. 7b). Collagen IV appeared to label blood vessels, as well as other non-vessel labelling, which could represent fibrous collagen IV deposition post-ICH. Due to autofluorescence and the inability to distinguish ‘true’ vessel labelling from fibrous collagen IV within the lesion, quantification in the lesion core was not performed. Quantification of various vessel parameters was performed at the border of the lesion, and no significant differences between the vehicle and hydrogel groups at 8 weeks were found in vessel area (Fig. 7c), vessel length density (Fig. 7d) or number of vessel branch points (Fig. 7e).

### Intracerebral Haemorrhage Results in Prolonged Changes in Inflammatory and Phagocytic Pathways That Are Not Affected by the Alpha2 Hydrogel

**Fig. 8:**
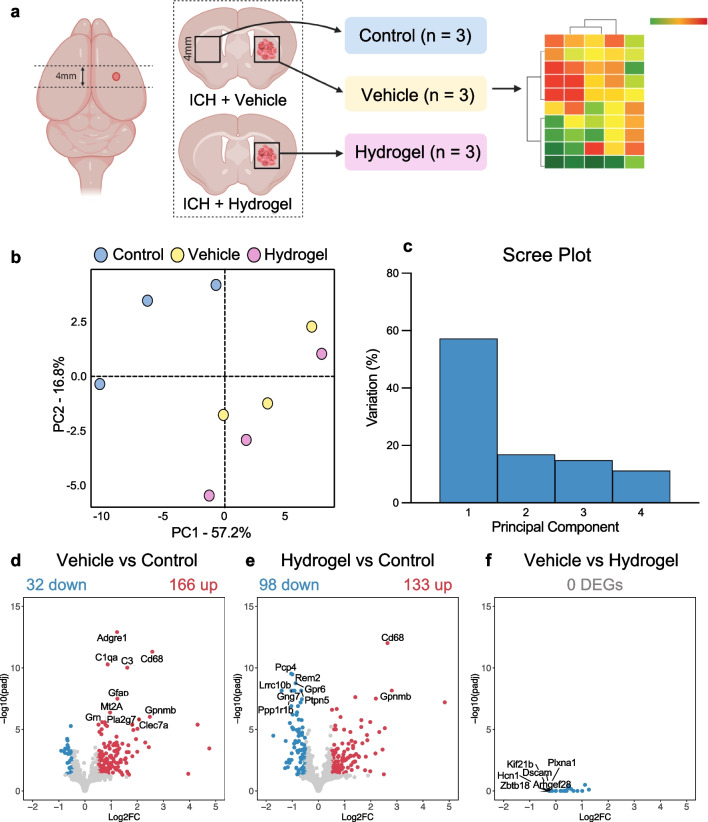
The change in gene expression seen 8 weeks after intracerebral haemorrhage is not altered by hydrogel administration. **a** Schematic showing the area of dissected tissue used for RNA-seq after intracerebral haemorrhage (ICH). A 4-mm cube of tissue comprising the majority of the striatum (haemorrhaged area) was excised from vehicle and hydrogel animals (*n* = 3/group). Tissue from the contralateral striatum of vehicle animals was taken as control ‘naïve’ tissue (*n* = 3). **b** Principal components analysis (PCA) of PC1 vs PC2. The first two PCs accounted for 74% of the variance between samples. Control samples cluster separately, whereas vehicle and hydrogel-injected samples cluster together. **c** Scree plot illustrating the contribution of the first four PCs to total variance. PC1 explained the majority of the variance between samples. **d**–**f** Volcano plots showing the upregulated (red) and downregulated (blue) differentially expressed genes (DEGs) in **d** vehicle vs control, **e** hydrogel vs control and **f** vehicle vs hydrogel comparisons. Cutoffs of *p* < 0.05 and log2 fold-change of 0.5 (either direction) were applied. The top 10 most significant DEGs are labelled. There were no DEGs in the vehicle vs hydrogel comparison

To assess long-term gene expression changes arising from ICH and to determine how these were affected by hydrogel administration, RNAseq was performed on brain tissue from the striatal (haemorrhage) region of both experimental groups, and of the contralateral striatum of the vehicle group (control) (Fig. 8a). Principal component analysis (PCA) revealed that while there was the spatial separation of control group samples from the other groups along PC1, and to a lesser degree along PC2, there was a minimal separation between vehicle and hydrogel samples on either PC (Fig. 8b). The scree plot of the first four PCs showed that PC1 accounted for the majority (57.2%) of the variation in the data, PC2 accounted for 16.8% and PC3 and PC4 made up the remainder of the variation (Fig. 8c).

Differential expression analysis was performed, using a FDR-padj of < 0.05 and a log2 fold change of > 0.5 in either direction to define DEGs. There were 198 DEGs in the vehicle versus control comparison, of which 32 were downregulated and 166 were upregulated (Fig. 8d). The top ten most significantly changed genes were *Adgre1*, *Cd68*, *C1qa*, *C3*, *Gfap*, *Mt2A*, *Gpnmb*, *Clec7a*, *Pla2g7* and *Grn*. For the hydrogel versus control comparison, there were 231 DEGs, with 98 downregulated and 133 upregulated genes. The top ten most significantly changed genes were *Penk*, *Cd68*, *Pcp4*, *Rem2*, *Gpr6*, *Gpnmb*, *Ptpn5*, *Lrrc10b*, *Ppp1r1b* and *Gng7* (Fig. 8e). For comparison of vehicle and hydrogel, there were no DEGs (Fig. 8f).

**Fig. 9:**
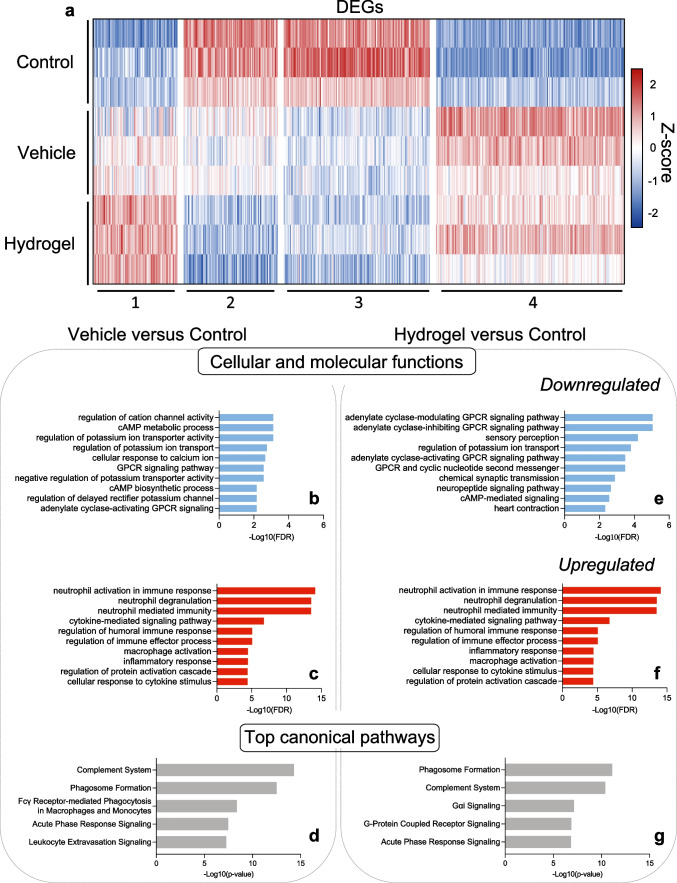
Gene-set enrichment and pathway analysis revealed that inflammatory processes were still altered 8 weeks after intracerebral haemorrhage. **a** K-means clustered heat map of differentially expressed genes (DEGs) between all experimental groups 8 weeks after intracerebral haemorrhage (ICH); four clusters were identified. **b**–**g** Gene set enrichment (EnrichR) was used to infer the top 10 **b** and **e** downregulated and **c** and **f** upregulated cellular and molecular functions in **b**–**d** vehicle versus control and **e**–**g** hydrogel vs control comparisons. **d** and **g** Pathway analysis was performed to identify the top canonical pathways linked to the DEGs in each comparison. Top canonical pathways involve both the up- and downregulated genes

Further analysis of the DEGs was performed to determine what functions and pathways were up- or downregulated in ICH tissue, compared to control tissue. Firstly, the DEGs in all comparisons were *k*-means clustered revealing four clusters (Fig. 9a). Clusters 1 and 4, and 2 and 3 were made up of genes that were up- or downregulated respectively in the vehicle and hydrogel groups, compared to control.

To explore whether the differences in gene expression between the vehicle and hydrogel samples versus control tissue were related to particular cellular and molecular functions, gene enrichment and pathway analysis were performed, and the top 10 enriched (up- and downregulated) processes were illustrated (Fig. 9b–g). Genes that were downregulated in vehicle versus control were related to gene ontology (GO) processes including ‘regulation of cation channel activity (GO:2001257)’, ‘cAMP metabolic process (GO:0046058)’ and ‘GPCR signaling pathways (GO:0007188)’ (Fig. 9b), and upregulated genes were involved in several processes relating to inflammation including ‘neutrophil mediated immunity (GO:0002446)’, ‘cytokine-mediated signaling pathway (GO:0019221)’ and ‘macrophage activation (GO:0042116)’ (Fig. 9c). The upregulated genes in the hydrogel versus control comparison (Fig. 9f) were almost identical to those in the vehicle comparison. However, some of the downregulated GO processes were different from the vehicle, including ‘chemical synaptic transmission (GO:0007268)’ and ‘neuropeptide signaling pathway (GO:0007218)’ (Fig. 9e).

IPA was performed to infer which canonical pathways were responsible for the DEGs in each group-wise comparison. The top 5 pathways linked to gene expression changes in vehicle and hydrogel versus control samples were identified (Fig. 9d and g). Consistent with the cellular and molecular functions identified by gene enrichment, the top canonical pathways in both groups related to aspects of inflammation including ‘phagosome formation’, ‘complement system’, ‘leukocyte extravasation’ and ‘acute phase response signaling’. This suggests that even at 8 weeks post-ICH, the predominant cellular functions in the ICH lesion relate to ongoing inflammation and phagocytosis and that these were not affected by the hydrogel.

## Discussion

This study aimed to assess the feasibility and safety of the novel hydrogel Alpha2 for application in CNS regeneration and to determine any potential therapeutic effects. Overall, when injected 7 days after ICH, the unfunctionalised Alpha2 hydrogel had neutral effects on functional outcomes and did not alter the gene expression profile in the affected tissues but did lead to a greater number of proliferating cells in the lesion. Taken together, the results confirm that Alpha2 is feasible and safe for application in brain repair.

The Alpha2 hydrogel was found to be injectable through clinically relevant gauge needles, which is essential for minimally invasive delivery to deep brain regions [19], and was able to fill the irregular shape of the cavity formed after ICH, becoming contiguous with the surrounding tissue, and estimations of brain tissue elasticity (stiffness) showed the hydrogel to be softer than that of brain tissue [37–39]. The hydrogel remained in situ for at least 2 weeks after injection but appeared to be degraded by 8 weeks. To fully evaluate the time course of hydrogel retention and degradation in the brain, future work could make use of the recently developed labelled Alpha2 and in vivo non-invasive imaging that would allow longitudinal assessment in the same animal [40, 41]. Although the exact timeline for regenerative events after ICH is unclear, angiogenesis and neurogenesis are thought to commence in the sub-acute to chronic phase after an acute brain injury and continue for several weeks or months thereafter [42–44]. Therefore the ideal hydrogel should remain in situ for at least several weeks to facilitate these processes but should also eventually be degraded, to be replaced by native tissue [19].

The hydrogel also appeared to provide a permissive microenvironment for host cell infiltration, which started as early as 24 h post-injection and continued for over 2 weeks. Several previous reports using unfunctionalised hydrogels in ICH models found very little material-tissue interaction and host cell infiltration, even at much later time points after injection [14, 15]. Some studies have reported significant immune cell infiltration after hydrogel implantation; however, this is likely to be more pronounced in studies using animal-derived hydrogels, which have known immunogenic properties, versus non-animal-derived hydrogels such as Alpha2 [45]. This potential non-immunogenic feature of Alpha2 is an advantage when translating its use in humans. A key concern in the design of biomaterials for application in the brain is matrix elasticity mismatch, which can exacerbate glial scarring [6, 46]. In this work, Alpha2 hydrogel administration did not affect glial scarring or cause a change in expression of the macrophage/microglia marker Iba1 at the lesion border compared with vehicle-injected controls, which together indicate that the material was well-tolerated and perhaps non-immunogenic. The potential non-immunogenicity of Alpha2 is supported by in vitro evidence, in which the inflammatory profile of mouse monocytes encapsulated within the hydrogel was studied. The authors reported no significant increase in the production of several pro-inflammatory cytokines, after 48 or 72 h of culture, indicating that the hydrogel did not elicit a pro-inflammatory response [20]. Overall, these data indicate that the Alpha2 hydrogel appeared to fulfil several of the features of an ideal hydrogel for brain repair.

Prior to hydrogel administration, there was no difference in haematoma volume between groups and 8 weeks later the hydrogel had no influence on the lesion volume or tissue atrophy. This is in contrast to a previous study that found reduced atrophy when a hyaluronic acid-based hydrogel was administered 1 week after ICH, which may represent a regenerative effect [47]. A keratin-based hydrogel encapsulated with an iron chelator administered acutely after ICH also led to reduced tissue atrophy [48], although this may be attributable to the protective effect of the iron chelator. However, data in this study indicates that the Alpha2 hydrogel did not exacerbate or improve the amount of tissue lost after ICH.

To confirm whether the hydrogel had any effect on functional outcomes, body weight monitoring and functional tests covering different domains of post-ICH deficits were performed. No effect on body weight, neuroscore or performance in the corner and cylinder tests was detected over 8 weeks after ICH. The lack of an effect on body weight, an indicator of welfare and ICH severity, suggests there were no major adverse effects or acute phase response to hydrogel injection [49, 50]. Similarly, a study by Ghuman and colleagues found that an ECM-based hydrogel administered at a chronic time point after ischaemic stroke was retained for several weeks and allowed host cell infiltration but had neutral effects on a battery of functional tests [51]. However, some studies using alternative hydrogels have reported a beneficial effect on the behavioural impairments seen in the neuroscore and corner test after ICH [12, 48, 52–54], although functional benefits are sometimes seen only when the hydrogel is combined with other factors, such as growth factors ([54]).

A key aim of regenerative medicine is to facilitate and enhance endogenous regenerative processes, such as angiogenesis and neurogenesis, that are typically halted by the biochemical and biophysical conditions in the damaged tissue. Using collagen IV as a marker of the basement membrane of blood vessels, there appeared to be an upregulation of collagen IV labelling within the core of the lesion, both with and without hydrogel treatment. However, quantification of blood vessel parameters at the lesion rim revealed no effect of the hydrogel on vessel area, density or number of branchpoints compared to the contralateral hemisphere, at 8 weeks after ICH. These data suggest that the hydrogel had no effect on the extent of vascularisation after ICH, although collagen IV may only label mature vessels and may not have detected nascent, actively developing blood vessels. However, injection of the Alpha2 hydrogel led to significantly more proliferative cells (positive for Ki67) within the lesion core. Ki67-expressing cells appeared to be positive for DCX, indicating the presence of proliferating neural progenitors in the lesion. Several reports using hydrogels in ICH have studied the expression of neurogenic markers [15, 52, 54, 55]. Interestingly, Lim and colleagues found a significant increase in the number of nestin-positive neural stem cells, after delivery of a EGF and gelatin-hydrogel combination therapy, but no increase with the hydrogel scaffold alone [54]. Similarly, another report found a significant increase in BrdU-positive cells with a hydrogel encapsulated with mesenchymal stem cells but not with the hydrogel alone [48]. Consequently, the significant increase in proliferative cells after administration of the Alpha2 hydrogel alone in the current work suggests that the biomaterial can support the proliferation of new cells. Labelling of β-III tubulin, a marker of immature neurones, uncovered elaborate networks of neuronal microtubules within the lesion itself in both experimental groups. Overall, these data suggest that the chronic ICH lesion is richly populated with not only Iba1-positive macrophages, but also a range of other cell types such as astrocytes, neuroblasts and immature neurones, together with the basement membrane protein, collagen IV. This is promising for the regenerative medicine approach, as it demonstrates that a number of cell types important for regeneration can enter the damaged tissue—a response which could be amplified with a targeted adjunct therapy.

RNA-seq was performed to study whether the hydrogel had an effect on the transcriptional profile after ICH. Several up- and downregulated genes were identified 8 weeks after ICH in both hydrogel and vehicle-injected tissue, but there were no changes in the DEGs between groups, supporting the idea that the material is safe but perhaps, largely functionally inert. Gene set enrichment and pathway analysis found that in both the hydrogel and vehicle-injected groups, DEGs were involved in pathways related predominantly to inflammation, such as neutrophil-mediated immunity, macrophage activation and cytokine-mediated signalling pathways. The top upregulated cellular and molecular functions identified in both experimental groups in the current work suggest that even as late as 8 weeks, inflammation is the dominant process occurring in the ICH lesion. However, the expression of some myeloid-related genes, such as *Cd68* and *Clec7a*, could be associated with a reparative immune cell phenotype. In addition, the canonical inflammatory interleukins are conspicuously absent from the list of DEGs in both groups, which is not typical of acutely inflamed tissue. Nevertheless, despite the presence of some DEGs associated with tissue repair and regeneration (*Hmox1*, *Dcx*, *Gfap*) in both groups, gene enrichment and pathway analysis did not identify pathways related to the either angiogenesis or neurogenesis, indicating that active repair is not a dominant process in lesioned tissue at 8 weeks.

Overall, this study is the first characterisation of the effects of the novel SAPH (Alpha2) hydrogel in the CNS. In testing in an experimental rat model of ICH, the hydrogel had several suitable properties for application in the brain, and when injected in vivo, the material was found to be safe, well-tolerated and could support the infiltration of multiple cell types. Furthermore, hydrogel injection had no negative effect on functional outcomes but did lead to an increase in the number of proliferating cells in the ICH lesion. These results indicate that the Alpha2 hydrogel may have potential as a delivery platform for other pro-regenerative therapies, as the retention and degradation rate may allow for sustained and targeted release of the therapy over time to enable regeneration after ICH.

## Data Availability

The supporting data of this study are available on request from the corresponding author.

## References

[1] Carmichael ST. Cellular and molecular mechanisms of neural repair after stroke: making waves. Ann Neurol. 2006;59(5):735–42. 10.1002/ana.20845. 16634041 10.1002/ana.20845

[2] Huang L, Wu ZB, Zhuge Q, Zheng W, Shao B, Wang B, et al. Glial scar formation occurs in the human brain after ischemic stroke. Int J Med Sci. 2014;11(4):344–8. 10.7150/ijms.8140. 24578611 10.7150/ijms.8140 PMC3936028

[3] Ohab JJ, Fleming S, Blesch A, Carmichael ST. A neurovascular niche for neurogenesis after stroke. J Neurosci. 2006;26(50):13007–16. 10.1523/JNEUROSCI.4323-06.2006. 17167090 10.1523/JNEUROSCI.4323-06.2006 PMC6674957

[4] Nih LR, Sideris E, Carmichael ST, Segura T. Injection of microporous annealing particle (MAP) hydrogels in the stroke cavity reduces gliosis and inflammation and promotes NPC migration to the Lesion. Adv Mater. 2017;29(32). 10.1002/adma.201606471. 10.1002/adma.201606471 PMC5595584 28650574

[5] Gonzalez-Nieto D, Fernandez-Garcia L, Perez-Rigueiro J, Guinea GV, Panetsos F. Hydrogels-assisted cell engraftment for repairing the stroke-damaged brain: chimera or reality. Polymers (Basel). 2018;10(2). 10.3390/polym10020184. 10.3390/polym10020184 PMC6415003 30966220

[6] Lam J, Lowry WE, Carmichael ST, Segura T. Delivery of iPS-NPCs to the stroke cavity within a hyaluronic acid matrix promotes the differentiation of transplanted cells. Adv Funct Mater. 2014;24(44):7053–62. 10.1002/adfm.201401483. 26213530 10.1002/adfm.201401483 PMC4512237

[7] Nih LR, Gojgini S, Carmichael ST, Segura T. Dual-function injectable angiogenic biomaterial for the repair of brain tissue following stroke. Nat Mater. 2018;17(7):642–51. 10.1038/s41563-018-0083-8. 29784996 10.1038/s41563-018-0083-8 PMC6019573

[8] Zhong J, Chan A, Morad L, Kornblum HI, Fan G, Carmichael ST. Hydrogel matrix to support stem cell survival after brain transplantation in stroke. Neurorehabil Neural Repair. 2010;24(7):636–44. 10.1177/1545968310361958. 20424193 10.1177/1545968310361958 PMC4697440

[9] Gelain F, Horii A, Zhang S. Designer self-assembling peptide scaffolds for 3-d tissue cell cultures and regenerative medicine. Macromol Biosci. 2007;7(5):544–51. 10.1002/mabi.200700033. 17477441 10.1002/mabi.200700033

[10] Purvis EM, O’Donnell JC, Chen HI, Cullen DK. Tissue engineering and biomaterial strategies to elicit endogenous neuronal replacement in the brain. Front Neurol. 2020;11:344. 10.3389/fneur.2020.00344. 32411087 10.3389/fneur.2020.00344 PMC7199479

[11] Emerich DF, Silva E, Ali O, Mooney D, Bell W, Yu SJ, et al. Injectable VEGF hydrogels produce near complete neurological and anatomical protection following cerebral ischemia in rats. Cell Transplant. 2010;19(9):1063–71. 10.3727/096368910X498278. 20412616 10.3727/096368910X498278

[12] Luo T, Guo T, Yang Q, Hao S, Wang J, Cheng Z, et al. In situ hydrogels enhancing postoperative functional recovery by reducing iron overload after intracerebral haemorrhage. Int J Pharm. 2017;534(1–2):179–89. 10.1016/j.ijpharm.2017.10.010. 28987454 10.1016/j.ijpharm.2017.10.010

[13] Thomas JM, Louca I, Bolan F, Sava OR, Allan SM, Lawrence CB, et al. Regenerative potential of hydrogels for intracerebral hemorrhage: lessons from ischemic stroke and traumatic brain injury research. Adv Healthc Mater. 2021;10(16):e2100455. 10.1002/adhm.202100455. 34197036 10.1002/adhm.202100455 PMC11468990

[14] Sang LY, Liang YX, Li Y, Wong WM, Tay DK, So KF, et al. A self-assembling nanomaterial reduces acute brain injury and enhances functional recovery in a rat model of intracerebral hemorrhage. Nanomedicine. 2015;11(3):611–20. 10.1016/j.nano.2014.05.012. 24907463 10.1016/j.nano.2014.05.012

[15] Zhang N, Luo Y, He L, Zhou L, Wu W. A self-assembly peptide nanofibrous scaffold reduces inflammatory response and promotes functional recovery in a mouse model of intracerebral hemorrhage. Nanomedicine. 2016;12(5):1205–17. 10.1016/j.nano.2015.12.387. 26772423 10.1016/j.nano.2015.12.387

[16] Kornev VA, Grebenik EA, Solovieva AB, Dmitriev RI, Timashev PS. Hydrogel-assisted neuroregeneration approaches towards brain injury therapy: a state-of-the-art review. Comput Struct Biotechnol J. 2018;16:488–502. 10.1016/j.csbj.2018.10.011. 30455858 10.1016/j.csbj.2018.10.011 PMC6232648

[17] Vedadghavami A, Minooei F, Mohammadi MH, Khetani S, Rezaei Kolahchi A, Mashayekhan S, et al. Manufacturing of hydrogel biomaterials with controlled mechanical properties for tissue engineering applications. Acta Biomater. 2017;62:42–63. 10.1016/j.actbio.2017.07.028. 28736220 10.1016/j.actbio.2017.07.028

[18] Morris O, Elsawy MA, Fairclough M, Williams KJ, McMahon A, Grigg J, et al. In vivo characterisation of a therapeutically relevant self-assembling (18) F-labelled beta-sheet forming peptide and its hydrogel using positron emission tomography. J Labelled Comp Radiopharm. 2017;60(10):481–8. 10.1002/jlcr.3534. 28623878 10.1002/jlcr.3534 PMC5601235

[19] Peressotti S, Koehl GE, Goding JA, Green RA. Self-assembling hydrogel structures for neural tissue repair. ACS Biomater Sci Eng. 2021;7(9):4136–63. 10.1021/acsbiomaterials.1c00030. 33780230 10.1021/acsbiomaterials.1c00030 PMC8441975

[20] Markey A, Workman VL, Bruce IA, Woolford TJ, Derby B, Miller AF, et al. Peptide hydrogel in vitro non-inflammatory potential. J Pept Sci. 2017;23(2):148–54. 10.1002/psc.2940. 27990715 10.1002/psc.2940 PMC5324702

[21] Burgess KA, Frati C, Meade K, Gao J, Castillo Diaz L, Madeddu D, et al. Functionalised peptide hydrogel for the delivery of cardiac progenitor cells. Mater Sci Eng C Mater Biol Appl. 2021;119:111539. 10.1016/j.msec.2020.111539. 33321610 10.1016/j.msec.2020.111539

[22] Faroni A, Workman VL, Saiani A, Reid AJ. Self-assembling peptide hydrogel matrices improve the neurotrophic potential of human adipose-derived stem cells. Adv Healthc Mater. 2019;8(17):e1900410. 10.1002/adhm.201900410. 31348622 10.1002/adhm.201900410

[23] Kilkenny C, Browne WJ, Cuthill IC, Emerson M, Altman DG. Improving bioscience research reporting: the ARRIVE guidelines for reporting animal research. In Public Libr Sci. 2010;8:e1000412. 10.1371/journal.pbio.1000412 PMC2893951 20613859

[24] Rosenberg GA, Mun-Bryce S, Wesley M, Kornfeld M. Collagenase-induced intracerebral hemorrhage in rats. Stroke. 1990;21(5):801–7. 10.1161/01.str.21.5.801. 2160142 10.1161/01.str.21.5.801

[25] Paxinos G, Watson C. The rat brain in stereotaxic coordinates. 6th ed. Boston: Academic Press/Elsevier, Amsterdam; 2007.

[26] Allred RP, Cappellini CH, Jones TA. The “good” limb makes the “bad” limb worse: experience-dependent interhemispheric disruption of functional outcome after cortical infarcts in rats. Behav Neurosci. 2010;124(1):124–32. 10.1037/a0018457. 20141287 10.1037/a0018457 PMC4888870

[27] Lekic TRW, Manaenko A, Fathali N, Zhang JH. Corner turning test for evaluation of asymmetry after intracerebral hemorrhage. In: Chen J, editor. Rodents Animal Models of Acute Neurological Injuries II. New York: Humana Press; 2012. p. 679–83.

[28] Feldman AT, Wolfe D. Tissue processing and hematoxylin and eosin staining. Methods Mol Biol. 2014;1180:31–43. 10.1007/978-1-4939-1050-2_3. 25015141 10.1007/978-1-4939-1050-2_3

[29] Bolger AM, Lohse M, Usadel B. Trimmomatic: a flexible trimmer for Illumina sequence data. Bioinformatics. 2014;30(15):2114–20. 10.1093/bioinformatics/btu170. 24695404 10.1093/bioinformatics/btu170 PMC4103590

[30] Kent WJ, Sugnet CW, Furey TS, Roskin KM, Pringle TH, Zahler AM, et al. The human genome browser at UCSC. Genome Res. 2002;12(6):996–1006. 10.1101/gr.229102. 12045153 10.1101/gr.229102 PMC186604

[31] Dobin A, Davis CA, Schlesinger F, Drenkow J, Zaleski C, Jha S, et al. STAR: ultrafast universal RNA-seq aligner. Bioinformatics. 2013;29(1):15–21. 10.1093/bioinformatics/bts635. 23104886 10.1093/bioinformatics/bts635 PMC3530905

[32] Howe KL, Achuthan P, Allen J, Allen J, Alvarez-Jarreta J, Amode MR, et al. Ensembl 2021. Nucleic Acids Res. 2021;49(D1):D884–91. 10.1093/nar/gkaa942. 33137190 10.1093/nar/gkaa942 PMC7778975

[33] Love MI, Huber W, Anders S. Moderated estimation of fold change and dispersion for RNA-seq data with DESeq2. Genome Biol. 2014;15(12):550. 10.1186/s13059-014-0550-8. 25516281 10.1186/s13059-014-0550-8 PMC4302049

[34] Zhu A, Ibrahim JG, Love MI. Heavy-tailed prior distributions for sequence count data: removing the noise and preserving large differences. Bioinformatics. 2019;35(12):2084–92. 10.1093/bioinformatics/bty895. 30395178 10.1093/bioinformatics/bty895 PMC6581436

[35] Nainggolan R, Perangin-angin R, Simarmata E, Tarigan AF. Improved the performance of the K-means cluster using the sum of squared error (SSE) optimized by using the Elbow Method. J Phys: Conf Ser. 2019;1361(1):012015. 10.1088/1742-6596/1361/1/012015.

[36] Xie Z, Bailey A, Kuleshov MV, Clarke DJB, Evangelista JE, Jenkins SL, et al. Gene set knowledge discovery with enrichr. Curr Protoc. 2021;1(3):e90. 10.1002/cpz1.90. 33780170 10.1002/cpz1.90 PMC8152575

[37] Flanagan LA, Ju YE, Marg B, Osterfield M, Janmey PA. Neurite branching on deformable substrates. Neuroreport. 2002;13(18):2411–5. 10.1097/00001756-200212200-00007. 12499839 10.1097/00001756-200212200-00007 PMC2408859

[38] Lampe KJ, Mooney RG, Bjugstad KB, Mahoney MJ. Effect of macromer weight percent on neural cell growth in 2D and 3D nondegradable PEG hydrogel culture. J Biomed Mater Res A. 2010;94(4):1162–71. 10.1002/jbm.a.32787. 20694983 10.1002/jbm.a.32787

[39] Lopez-Fagundo C, Bar-Kochba E, Livi LL, Hoffman-Kim D. Franck C Three-dimensional traction forces of Schwann cells on compliant substrates. J R Soc Interface. 2014;11(97):20140247. 10.1098/rsif.2014.0247. 24872498 10.1098/rsif.2014.0247 PMC4208357

[40] Liang Y, Bar-Shir A, Song X, Gilad AA, Walczak P, Bulte JW. Label-free imaging of gelatin-containing hydrogel scaffolds. Biomaterials. 2015;42:144–50. 10.1016/j.biomaterials.2014.11.050. 25542802 10.1016/j.biomaterials.2014.11.050 PMC4279110

[41] Piejko M, Walczak P, Li X, Bulte JWM, Janowski M. In vitro assessment of fluorine nanoemulsion-labeled hyaluronan-based hydrogels for precise intrathecal transplantation of glial-restricted precursors. Mol Imaging Biol. 2019;21(6):1071–8. 10.1007/s11307-019-01341-6. 30850968 10.1007/s11307-019-01341-6 PMC6733661

[42] Liu CZ, Zhou HJ, Zhong JH, Tang T, Cui HJ, Zhou JH, et al. Leukemia inhibitory factor decreases neurogenesis and angiogenesis in a rat model of intracerebral hemorrhage. Curr Med Sci. 2019;39(2):298–304. 10.1007/s11596-019-2034-2. 31016525 10.1007/s11596-019-2034-2

[43] Yang S, Song S, Hua Y, Nakamura T, Keep RF, Xi G. Effects of thrombin on neurogenesis after intracerebral hemorrhage. Stroke. 2008;39(7):2079–84. 10.1161/STROKEAHA.107.508911. 18436875 10.1161/STROKEAHA.107.508911

[44] Zhou HJ, Tang T, Cui HJ, Yang AL, Luo JK, Lin Y, et al. Thrombin-triggered angiogenesis in rat brains following experimental intracerebral hemorrhage. J Neurosurg. 2012;117(5):920–8. 10.3171/2012.8.JNS112152. 22957530 10.3171/2012.8.JNS112152

[45] Modo M, Ghuman H, Azar R, Krafty R, Badylak SF, Hitchens TK. Mapping the acute time course of immune cell infiltration into an ECM hydrogel in a rat model of stroke using (19)F MRI. Biomaterials. 2022;282:121386. 10.1016/j.biomaterials.2022.121386. 35093825 10.1016/j.biomaterials.2022.121386

[46] Maclean FL, Horne MK, Williams RJ, Nisbet DR. Review: biomaterial systems to resolve brain inflammation after traumatic injury. APL Bioeng. 2018;2(2):021502. 10.1063/1.5023709. 31069296 10.1063/1.5023709 PMC6481708

[47] Liu Y, Hsu YH, Huang AP, Hsu SH. Semi-interpenetrating polymer network of hyaluronan and chitosan self-healing hydrogels for central nervous system repair. ACS Appl Mater Interfaces. 2020;12(36):40108–20. 10.1021/acsami.0c11433. 32808527 10.1021/acsami.0c11433

[48] Gong Y, Wang Y, Qu Q, Hou Z, Guo T, Xu Y, et al. Nanoparticle encapsulated core-shell hydrogel for on-site BMSCs delivery protects from iron overload and enhances functional recovery. J Control Release. 2020;320:381–91. 10.1016/j.jconrel.2020.01.029. 31972243 10.1016/j.jconrel.2020.01.029

[49] Lyoumi S, Tamion F, Petit J, Dechelotte P, Dauguet C, Scotte M, et al. Induction and modulation of acute-phase response by protein malnutrition in rats: comparative effect of systemic and localized inflammation on interleukin-6 and acute-phase protein synthesis. J Nutr. 1998;128(2):166–74. 10.1093/jn/128.2.166. 9446838 10.1093/jn/128.2.166

[50] Talbot SR, Biernot S, Bleich A, van Dijk RM, Ernst L, Hager C, et al. Defining body-weight reduction as a humane endpoint: a critical appraisal. Lab Anim. 2020;54(1):99–110. 10.1177/0023677219883319. 31665969 10.1177/0023677219883319

[51] Ghuman H, Gerwig M, Nicholls FJ, Liu JR, Donnelly J, Badylak SF, et al. Long-term retention of ECM hydrogel after implantation into a sub-acute stroke cavity reduces lesion volume. Acta Biomater. 2017;63:50–63. 10.1016/j.actbio.2017.09.011. 28917705 10.1016/j.actbio.2017.09.011 PMC5653430

[52] Xu J, Duan Z, Qi X, Ou Y, Guo X, Zi L, et al. Injectable gelatin hydrogel suppresses inflammation and enhances functional recovery in a mouse model of intracerebral hemorrhage. Front Bioeng Biotechnol. 2020;8:785. 10.3389/fbioe.2020.00785. 32760708 10.3389/fbioe.2020.00785 PMC7371925

[53] Zhu Q, Gong Y, Guo T, Deng J, Ji J, Wang B, et al. Thermo-sensitive keratin hydrogel against iron-induced brain injury after experimental intracerebral hemorrhage. Int J Pharm. 2019;566:342–51. 10.1016/j.ijpharm.2019.05.076. 31158456 10.1016/j.ijpharm.2019.05.076

[54] Lim TC, Mandeville E, Weng D, Wang LS, Kurisawa M, Leite-Morris K, et al. Hydrogel-based therapy for brain repair after intracerebral hemorrhage. Transl Stroke Res. 2020;11(3):412–7. 10.1007/s12975-019-00721-y. 31432328 10.1007/s12975-019-00721-y

[55] He Y, Qu Q, Luo T, Gong Y, Hou Z, Deng J, et al. Human hair keratin hydrogels alleviate rebleeding after intracerebral hemorrhage in a rat model. ACS Biomater Sci Eng. 2019;5(2):1113–22. 10.1021/acsbiomaterials.8b01609. 33405801 10.1021/acsbiomaterials.8b01609

